# Investigating the Feasibility, Usability, and Efficacy of a Mobile App to Reduce Anxiety and Depression in Families of Critical Care Cancer Patients: A Quasi-Experimental Pilot Study

**DOI:** 10.3390/healthcare14030353

**Published:** 2026-01-30

**Authors:** Anthony Faiola, Saira Soroya, Reinhold Munker, Zhonglin Hao, Joshua Lambert

**Affiliations:** 1 College of Nursing, University of Cincinnati, Cincinnati, OH 45202, USA; 2 Department of Information and Library Science, College of Education, University of Southern Connecticut State University, Harford, CT 06515, USA; 3 Division of Hematology, Blood/Marrow Transplantation and Cellular Therapy Program, Markey Cancer Center, College of Medicine, University of Kentucky, Lexington, KY 40506, USA; 4 Markey Cancer Center, College of Medicine, University of Kentucky, Lexington, KY 40506, USA

**Keywords:** mHealth, families, cancer, intensive care unit, anxiety, depression

## Abstract

**Background**: Cancer patients admitted to the bone marrow transplant (BMT) unit face life-threatening medical conditions. Consequently, their family members experience uncertainty, resulting in high levels of anxiety and depression (AD). Limited updates and communication from medical staff exacerbate these emotional burdens. To address these challenges, we developed a mobile health (mHealth) intervention, FamCare*Plus*, and evaluated its feasibility, usability, and efficacy. We hypothesized that the FamCare*Plus* application would demonstrate a high degree of feasibility and usability and would reduce AD compared to a control group relying solely on traditional communication through the nurses’ station. **Methods**: We employed a quasi-experimental pretest/posttest non-randomized, non-blinded self-report design over 3 weeks, with an experimental group (*n* = 10) using FamCare*Plus* and a control group (*n* = 9). We selected participants via convenience sampling using the electronic medical record to identify eligible patients and families, guided by inclusion and exclusion criteria. We used descriptive statistics and the Hospital Anxiety and Depression Scale (HADS) guidelines to analyze the data. Feasibility was defined by a retention rate > 80%, with usability testing using the System Usability Scale (SUS) and NASA Task Load Index (NASA-TLX) surveys. The HADS measured AD, comparing baseline to Week 3. **Results**: We met our feasibility criteria of >80%. All SUS and NASA scores were in the higher index, suggesting a significant degree of usability and low workload demand on participants. For efficacy, we compared baseline mean scores, with the experimental group reporting lower AD levels at Week 1 (41.9% and 27.8%, respectively) than the control group (55.2% and 34.2%, respectively). From Week 1 to Week 3, the percentage change showed an 8.6% decrease in anxiety in the experimental group, compared to a 12.8% decrease in anxiety in the control group. These results were consistent when analyzed according to HADS guidelines. **Conclusions**: The findings of this study provide preliminary evidence that the FamCare*Plus* intervention is feasible and usable, while also demonstrating that its use may be associated with a sustained reduction in AD levels among family members of patients admitted to the BMT unit. These outcomes underscore the potential of digital interventions to address disparities in patient health information access and psychosocial support.

## 1. Introduction

When cancer patients are admitted to the intensive care unit (ICU) for inpatient bone marrow transplant (BMT) treatment, chemotherapy, or reconstructive surgery, their conditions are typically urgent. As a result, families [1,2] of patients receiving cancer treatment require timely medical updates, clear communication from staff, and proximity to their loved one [3]. When the needs of family members are met, there are desirable outcomes for cancer patients and their families. With the critical condition of these patients, most ICU clinicians’ (physicians and nurses) efforts are focused on stabilizing and preserving life as their chief responsibility, where family members’ needs may be of a lower priority in the overall plan of care [4,5]. Even under acceptable health care conditions, ICU patients and their families often experience uncertainty and heightened levels of confusion. Moreover, the devastating nature of a life-threatening condition has a profound effect on the mental health of family members, which inevitably produces increased psychological burden [6]. While the ICU is a critical-care facility dedicated to patients who need constant specialized bedside care [7], it remains an environment where family members often develop high levels of anxiety and depression (AD) [8,9,10].

Studies consistently demonstrate that families are more likely to develop anxiety, depression, post-traumatic stress disorder (PTSD) [11,12,13,14,15,16], and disruptions to family relationships [17,18] because of challenges surrounding less-than-optimal information flow from the bedside [19]. One study found that 54% of families had poor understanding of the patient’s diagnosis and treatment due to the lack of communication [19]. Other studies show that this poor understanding is due in part to a lack of timely medical updates and staff communication, especially if family members live in locations remote from the point of care [3,4,20]. For example, key factors affecting the mental health of family members of ICU patients include isolation from the patient [21,22,23,24,25,26,27,28], socioeconomic inequalities [29,30,31], and increased transportation needs due to inter-hospital transfers [32,33,34].

Additional studies show that one-third of people treated for cancer have a common mental health condition [35]. This includes depressive mental disorders, with rates that may be as much as three times higher than those in the general population [36]. These conditions affect cancer patients, as well as family members, particularly in the early post-treatment period, where there is a shift in focus from treatment and recovery to fear of recurrence and long-term side effects. In addition, these conditions significantly influence the well-being of family members during and after diagnosis and treatment, as well as the long-term health outcomes of the patient [37,38].

Researchers argue that during health care crises, when families are restricted from being physically present, their personal care becomes particularly important [39,40]. As a result, there is a need for increased communication between the ICU care team and family members, adopting “family-centered tools” that address restrictions on family access to patient-health updates and communication [41,42,43,44]. For example, during the COVID-19 pandemic, hospital visitation privileges were limited [45], and families encountered the suspension of ICU visitation rights and, as a result, experienced a high prevalence of acute stress disorder even 3 months after patient discharge [12]. Isolation due to the pandemic especially exacerbated the mental health of rural families as visiting restrictions increased. The results were catastrophic for racial and ethnic, geographic, and socioeconomic disadvantaged families.

Research shows that apart from the pandemic, rural communities (high on the isolation scale) [46] already experience social disconnectedness that correlates to poor mental health [47,48]. Compounded with this disparity factor, limited visitation rights only add to and result in families experiencing significant psychological dysfunction [26]. Family members experiencing negative emotions, such as guilt, hopelessness, and despair, require support from health care staff. Such support could improve care coordination between families and health care staff, especially when there is an exchange of information and regular health updates [49]. In addition to reducing mental trauma, research demonstrates that enhanced communication improves family and patient satisfaction, while advancing health outcomes [50].

Specifically, when family members are geographically separated because of long-term cancer treatment, they rely on smartphones to accommodate access to the patient or health updates. Consequently, rural families continue to face barriers in receiving and understanding patient information that would allow them to make informed decisions or to achieve a sense of closure at the time of the patient’s passing [28]. Although a majority of rural family members use smartphones, the greater challenge is related to their distant geographic location. Added to the weight of their socioeconomic condition, families of cancer patients must travel long distances to stay in close proximity to the bedside. Often, comfort is only found in knowing (hour by hour) what is happening with their loved one. This is especially true for older adults, who are limited financially [51].

In exploring ways to overcome these challenges, the first priority to consider is how to increase the flow of patient health updates to families from the bedside—not so much the quantity of information but the regularity and ease of access of information that is relevant to family members. Moreover, establishing rapport with members of the care team is imperative during this time of patient isolation and limited access to updates. Researchers suggest that hospital administrations should adapt “family-centered tools” to positively overcome restrictions on family access to patient health updates and communication [39].

Physicians have made attempts to support critical-care patient families through mobile health (mHealth) applications, with evidence that their use reduces family anxiety and increases early discharge and efficiency of care following discharge [52,53,54]. Such tools might include digital solutions that provide relevant, easily accessible information from the bedside. Such interventional support would be specifically designed to safely and securely communicate patient health and wellness information to families, providing the greatest benefit to their mental health [51,55,56]. Researchers further argue that providers and hospital administrators should explore mHealth as part of standardized care for families [57]. This includes digital technologies focused on communication and information flow [58,59] that have the potential to reduce the psychological burden on family members [60,61]. These applications would lead to solutions that result in higher patient and family satisfaction, more positive patient outcomes, and reduced AD [62].

Currently, ICU patients and their families use three types of digital tools to communicate and, if necessary, some family members are given access to patient health data. First, social media, email, and texting are the most common forms of communication that patients and families use to communicate with each other and with others outside the family. However, these digital tools do not provide any health data security and are not HIPAA compliant. Next, increasingly popular are health community applications that help patients and their families create public forums, where family and friends can receive updates. These applications provide dashboards that allow for scheduling visits, describing a health journey, and gathering information from the nursing staff via text and email. Last, some patients prefer the use of Epic MyChart. With proxy access, family members can get updated vitals, lab results, and medication lists. MyChart, however, rarely captures physician notes beyond vitals and disease-specific information and does not provide information about the patient’s bedside condition and general wellness (e.g., sleep quality, eating patterns, medication compliance). Moreover, MyChart provides personal health data that many patients and their families may not want to be made available to extended family or friends if they are granted proxy access [63,64,65,66].

Although many of these tools continue to provide considerable support to patients and families, their purpose is specific and does not provide the same digital support offered by the proposed mHealth app FamCare*Plus*. In addition, they do not focus on reducing the AD of family members. Although reduction in AD may be a positive outcome of their use, it is not a focal point of concern. As such, the particular family-centered health care need we address provides a balance of vitals and data updates on the patients’ health and wellness profile not found in the EMR, as well as the real-time condition of the patient, texting and video support, and quick access to social and mental health services. Although with the proposed application, we do not focus on creating a public community of care, we seek to make specific connections between one (remote) family member or a small group of family members and clinicians at the bedside.

Hence, to address the challenges noted, in this study, we intended to evaluate the feasibility, usability, and efficacy of a novel mHealth app intervention, referred to as FamCare*Plus* [67,68]. The aim of the intervention is to reduce cancer patient family AD by enhancing communication and coordination between families and clinicians at the bedside. This is achieved by increasing access to patient vitals and wellness information as well as connecting families to social and mental health services. Apart from its potential to reduce AD, FamCare*Plus* could prove invaluable for positively affecting the social determinants of health for rural families, while building trust and satisfaction with health care services.

As such, we hypothesized that after 3 weeks of using the FamCare*Plus* intervention (from baseline), https://apps.apple.com/us/app/famcare/id6446958621 (accessed on 20 January 2026), participants conveniently assigned to the app-based experimental group would demonstrate that a larger study using FamCare*Plus* was feasible and that the application was easy to learn and use and had a high potential to reduce AD when compared to the control group, which relied on traditional means of communication through the nurses’ station.

## 2. Methods

### 2.1. The Intervention

The FamCare*Plus* platform is a family-centered mHealth system intended to inform and incentivize cancer patient families to be proactive in family–clinician decision-making in the subsequent recovery of the patient. FamCare*Plus* functions as a supplement to traditional forms of communication with the BMT unit, giving cancer patient families increased transparency of bedside care. These benefits could have a significant influence on the mental health outcomes in families of cancer patients and indirectly affect patient recovery.

The principal investigator and his research team designed and developed the first FamCare*Plus* prototype from 2017 to 2020 [67], followed by conducting usability testing and interviews. Favorable user feedback resulted in the final product developed from 2020 to 2023, followed by additional usability testing, as outlined below. The FamCare*Plus* app went through multiple design iterations from 2017 to 2023, with input and usability testing with a broad array of clinicians from 2 major university health care systems, including nurses, physicians, physician assistants, health science students, and health care clergy.

**Figure 1:**
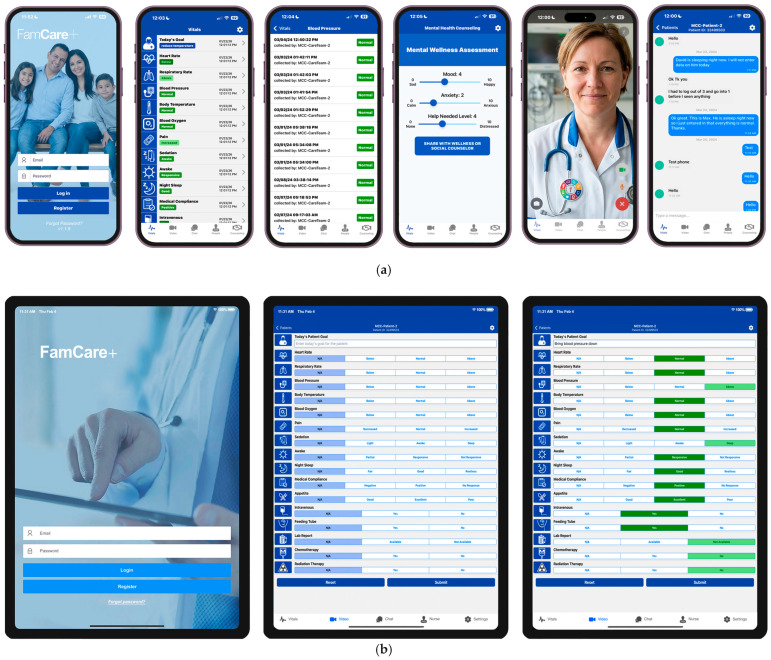

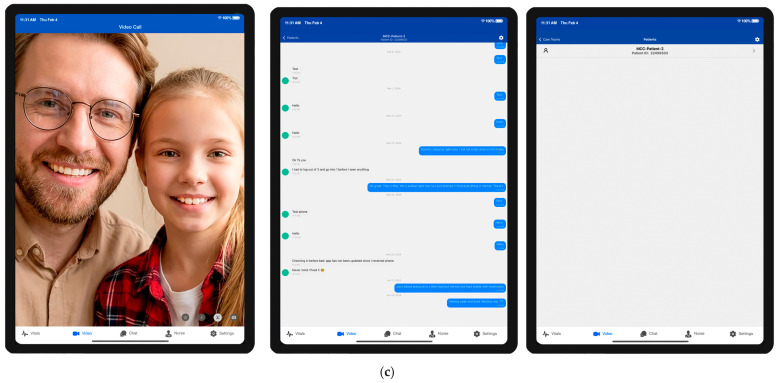
FamCare*Plus* interfaces for smartphone (for remote families) login, patient vitals and wellness measures reading, history timeline of specific vitals and wellness measure, mental wellness assessment tool, video conference with bedside, secure texting tool (**a**), FamCare*Plus* interfaces of tablet (for the bedside) Login, patient vitals and wellness measures input (at default), patient vitals and wellness measures input (after input) (**b**), and FamCare*Plus* interfaces of video conference (with families), secure chat tool (from bedside side), patient identified (e.g., shows only one patient (**c**).

FamCare*Plus* includes a mobile application used by families outside the clinical setting, usually in a distant, remote location. The application provides the following tools: vitals/wellness updates, video conferencing, texting, and a mental health self-assessment tool that relays outcome data to social or mental health services. FamCare*Plus* also includes an additional application for the clinical side, using a tablet at the bedside. This feature provides clinicians or family members in the room the ability to input real-time vitals/wellness updates in easy-to-understand qualitative measures for other family members in remote locations. FamCare*Plus* also includes texting and video conferencing for families (outside the hospital) to the patient’s bedside. See Figure 1 for examples of the FamCare*Plus* interfaces for login and data transfer to remote families.

After obtaining family member consent, the research team placed the FamCare*Plus* bedside tablet in the patient’s room in a location that would not interfere with patient care. At this time, the research team trained a family member on how to use the mobile application and the tablet application. Although FamCare*Plus* is currently in the Apple store, family members were provided an iPhone with the application already installed. In this way, we did not need to work through each participant’s personal phone settings. Although most family members participating in the study used iPhones, those with Android phones quickly learned how to use FamCare*Plus* without any issues. Data input into the tablet was done throughout the week by a research team member or a family member in the room. Because there were multiple family members visiting the patient, they often helped input data at the bedside. In this way, the family member who kept the iPhone provided by the study received the data. Finally, the nursing administrator did not allow bedside nurses to participate in the study. Given existing clinical burdens already placed on bedside nurses, adding the responsibility of the study was not deemed advantageous to their existing duties.

The FamCarePlus application was designed with a backend tracking system to record all user login activity and patient information input at the bedside. Regarding HIPAA compliance and personal health information safeguards, the FamCarePlus system uses encrypted code on a secure server when data are collected. However, it should be noted that no study participant or patient’s personal or identifiable health information is in any way input into the system during registration or throughout the study process. A randomized patient code is used to connect the smartphone to the bedside tablet in the cloud server with no connection or affiliation to the patient or any family member. This configuration was intentionally devised to avoid issues related to HIPAA policy.

### 2.2. Design

In this study, we employed a quasi-experimental [69], pretest–posttest, nonrandomized [70], and nonblinded self-reporting design to evaluate the effects of the mHealth application, FamCare*Plus*, on AD in family members of BMT patients during an inpatient 3-week treatment. We selected participants based on convenience sampling and suitability for the study [71], and we executed the study in the BMT Unit of Markey Cancer Center (MCC) at the University of Kentucky.

### 2.3. Participants

Study participants consisted of an experimental group of 10 families who used the FamCare*Plus* application, with the control group comprising 10 families who relied on traditional forms of communication through the nurses’ station, e.g., phone calling. The experimental group also had available to them access to the nurses’ station, if needed (i.e., they could use both the intervention and the nurses’ station).

Before contacting patients and family members, we received approval from the University of Kentucky Institutional Review Board and the MCC Protocol Review and Monitoring Committee, which reviews and approves all cancer human subjects research as well as all data auditing for quality control purposes. After receiving these approvals, the research team received approval from the ranking administrator of the MCC BMT unit. This approval allowed us a smooth, well-coordinated interaction with all the nurses who served multiple patients during multiple shifts throughout the week.

We recruited families of newly admitted patients to the BMT unit within three to five days after they arrived on the floor. This process began with the research team working with BMT physicians to gather information on daily incoming patients based on a review of electronic medical records (EMRs), per the inclusion and exclusion criteria. The physician researchers provided short lists of potential participants several times per week.

Once the patients’ names were provided, a member of the research team approached the family members and the patient when they were in the room together. The recruitment process included the use of a small color paper brochure that explained the intervention, followed by a review of the consent form.

Inclusion criteria were as follows: (1) BMT patients with prognoses that had a high degree of positive outcome, (2) patients who are 18 years and older, (3) patients with at least 1 adult family member able to provide consent for participation in the study for 4 weeks, (4) patients with experience using a smartphone, (5) patients who speak English, and (6) patients with at least one family member in a remote location who can receive information from the FamCare*Plus* system. Regarding inclusion point 6, remote location refers to family member participants who live approximately a 30-min drive or more from MCC. Given that most of Kentucky is rural, especially around Lexington, the majority of BMT patients and their family members are covered in this inclusion point. However, whether families live 30 min or 3 h (by car) from MCC, the sense of distance, lack of communication, and separation from the patient result in approximately the same psychological outcome.

Exclusion criteria were as follows: (1) patients who were not recently admitted to BMT (within the past week); (2) patients who were participating in other trials; and (3) family members who were unwilling to actively participate in inputting data into the FamCare*Plus* app, complete follow-up surveys, or provide contact information. Mental health prescreening of family-member participants was not an exclusion criterion—that is, an assessment of family member history of AD or other mental health impairments. We determined that identifying participants with preexisting or concomitant mental health disorders was not rational, given that all family members were experiencing some level of heightened AD at the time of the patient’s admittance to the MCC BMT unit. In other words, if we discovered (through a prescreening psychometric tool) that any family member had a history of mental health dysfunction, we would be unable to concisely isolate those causal effects from the existing event. As such, to identify or separate any family member with a history of adverse mental health would require a considerable degree of psychiatric assessment well beyond the scope of this pilot study.

We set our recruitment target at 20 (experimental and control) family members of individual patients admitted to the inpatient BMT facility of the MCC. Recruitment began on 21 July 2023 and ended on 22 March 2024.

### 2.4. Feasibility

The feasibility of our study depended on family members’ willingness to participate, given the health condition of the patient, their availability, and their general interest in contributing to scientific discovery [72]. Criteria for determining feasibility include recruitment rates and retention rates [73]. We (a priori) defined retention as >80% (>8 participants out of 10) able to complete the experimental arm in 3 weeks. Because the control arm is well established, that is, the use of the nurses’ station as the primary source for communication and information, a feasibility evaluation was not required. Retention is related to the frequency of participants completing the study, including the proportion of dropouts throughout the 3 weeks of data collection. We calculated the retention rate as the number of family members completing all 3 weeks of the study. The research team recorded all adverse events occurring throughout the study.

### 2.5. Usability

Post-measurement data collection included 2 usability measures, the System Usability Scale (SUS) and the NASA-TLX survey (NASA). We used the SUS to assess the perceived usability of the intervention, FamCare*Plus*. It is a validated, reliable scale consisting of 10 items that are rated on a 5-point Likert scale ranging from 0 to 4 [74]. The SUS quantifies the usability of products and services, including software, mobile apps, websites, or any interactive device with an interface. Three examples of questions include the following: (1) “Thought the system was easy to use,” (2) “Found the various functions in this system were well integrated,” and (3) “Thought there was too much inconsistency in this system” [75]. User response options range from 1 (Strongly Disagree) to 5 (Strongly Agree). Researchers used data from 241 SUS usability studies to create a curved grading scale, from which they found that the mean SUS score was 68, with 50% falling below and above it. A mean SUS score > 80 could be considered “good” as evidence of an above-average user experience, <70 could be considered as having usability issues. As such, the top and bottom 15 percentiles correspond to A and F grades, with further subdivisions for A+, A, A− and additional breakdowns for grades of B and C [76,77].

The NASA survey is a validated instrument designed for participants to assess the subjective workload of their use of the FamCare+ app [78,79]. The workload of a task depends on a variety of factors, such as the nature and difficulty of the task, as well as the aptitude and attitude of the individual. The NASA survey consists of 2 parts divided into 6 subjective subscales that are represented on a single page: Mental Demand, Physical Demand, Temporal Demand, Performance, Effort, and Frustration. Each NASA subscale is scored from 0 to 100 in 5-point increments. Although there is no standard NASA baseline, studies suggest that low workload scores are below 40, moderate workload scores are between 40 and 70, and high workload scores are above 70. Higher scores in each subscale indicate higher demand of the task and, in some cases, a lack of success in executing the task. Scores from all subscales were averaged for the total unweighted NASA score.

### 2.6. Efficacy

To determine efficacy, we conducted the self-reporting Hospital Anxiety and Depression Scale (HADS) neuropsychology survey [80]. We gave the HADS to family participants following recruitment and consent, and at the conclusion of 3 weeks of using the FamCare*Plus* intervention. The HADS includes 14 items on a 4-point Likert scale (range 0–3), with two 7-item subscales, 0–21 each, where higher scores indicate AD symptoms. As a widely used measure of patient mental health, the HADS [81] was designed as a screening tool to identify AD in nonpsychiatric clinical populations. The HADS has been used in a range of clinical contexts and conditions, including cancer [82], and it has been validated with chronic illness and cancer survivors and has strong reliability and validity [83].

Items are scored 0–3 (subscale range 0–21), with scores ≥ 8 categorized as borderline abnormal and ≥11 categorized as abnormal. Scores between 0 and 7 represent “no case,” 8 and 10 indicate “possible case,” and 11 and 21 suggest a “probable case of anxiety/depression.” These subscale range thresholds have been validated against clinical interviews with specificity and sensitivity at 0.80 [84]. Studies have reported acceptable internal consistency for both anxiety at 0.89 and depression at 0.86 subscales [81].

Participant family members were provided with the HADS immediately after the consenting process was completed to establish the Week 1 (or baseline) score for their current level of AD. Week 1 is the baseline. At the completion of the 3 weeks of using FamCare*Plus*, we collected the phone and tablet and asked the same family member to fill out the HADS once again for the post-study measure. This was followed by asking the family member to fill out the 2 usability measures, the SUS and the NASA.

### 2.7. Demographics

We collected baseline comparability demographic data, including gender, age, ethnicity, and the distance each family participant had to drive from their home to the MCC in Lexington, Kentucky. All family member participants were either a spouse or a parent. Given that this was a pilot study, we decided not to collect participant data related to education, annual income, current or prior mental health, or level of mobile application or technology acceptance, as might be measured using the technology acceptance model [85]. Although understanding differences in BMT patient acuity or prognosis may be an additional factor that could affect the degree of AD of each family member participating in the study, we determined that it was beyond the scope of this pilot study to identify or measure the complexity of each patient case. To do so would consist of a considerable review of each patient’s EMRs. In principle, all BMT patients are accepted into the BMT unit with aligned diagnoses. Although their prognoses may vary, determining how much prognosis affects family mental health would be broadly speculative.

### 2.8. Data Analysis

Because of the small sample size and experimental nature of this study, descriptive statistics were the primary means of data analysis. We also performed median tests on the differences between Week 1 and Week 3 exploratorily to inform potential improvements to the FamCare*Plus* intervention, as well as how future studies might be implemented. These estimates and their effect size will be used to inform future studies powered to investigate the efficacy of FamCare*Plus* as compared to a control group. We also used an independent *t*-test to illustrate the mean differences between the 2 groups for each question of HADS. We analyzed FamCare*Plus* backend data (used to track user login activity) using the Pearson correlation coefficient (r).

## 3. Results

### 3.1. Demographic Findings

Regarding gender, female participants were the majority participants in both groups, Experimental: F/M = 8/1 (88.88%/11.12%) and Control: F/M = 8/2 (80.00%/20.00%). We observed a good range in age of both groups participating in the study, with the majority being 40 years and older. Surprisingly, however, with both groups we saw five participants over the age of 70 years participating: experimental: two (2.20%) and control: three (30.00%). Regarding ethnicity, white participants were in the majority, with Experimental at 88% and Control at 90%. This ratio is reflected in the overall demographics of rural Kentucky, where African Americans are 7.88% of the population [86]. Last, we observed that the majority of the participants lived 25 to 250 miles from the MCC in Lexington, Kentucky. This included almost 90% of the Experimental group and 70% of the Control group (see Table 1).

### 3.2. Feasibility Findings

Although the recruitment process involved some challenges, we anticipated measures on recruitment, with good retention rates to meet the established threshold. During the 8 months of recruitment, our convenience sampling yielded approximately 1 in 4 families who were interested in speaking with us, of which 19 families agreed to participate in the study. These were families that were previously identified by the oncologist team member as potentially good candidates for the study. Hence, we obtained an enrollment of 25%.

Of the experimental group, only one family member withdrew from the study (1 week in). This left us with 9 families in the experimental group and 10 in the control group, hence a 5% attrition rate. As such, our >80% threshold was met. Although there were several minor technical issues with the mobile app, we recorded no adverse events from any of the participants in the experimental group.

### 3.3. Usability Findings

We analyzed SUS scores in two ways. Raw score ratings are shown in Table 2 for all participant family members for each question, with an overall mean score of 4.20 (SD = 0.84). Specific SUS questions are listed in Table 2. We also analyzed SUS scores to determine an overall satisfaction score according to the original criteria method [87]. The total mean SUS score of 80 (SD = 21.02) was above average, indicating good usability, with individual scores ranging from 57.5 to 100 (Table 3).

Regarding prior NASA testing, studies suggest that scores below 40 are considered low workload (see Table 4). All mean scores of our study were below the threshold of 40, indicating participants’ workload experience was considerably low demand while using the smartphone mobile application. This includes Frustration being the lowest score (17.14) and Effort being the highest (30.00). The overall mean score was (27.50), suggesting a low demand of tasking while using the intervention. Participants also reported high confidence, particularly in the Frustration workload demand, and in the overall use of the app, with a standard deviation of 17.14 and 15.45, respectively.

Only 7 of the 9 participants who completed the study filled out the SUS and NASA surveys. Two family members were not interested in participating beyond the HADS survey. However, according to usability researchers, this number of participants is more than sufficient to determine usability [88,89]. Although these usability findings are extremely positive, they are not conclusive and need further study.

### 3.4. Efficacy Findings

Before presenting the efficacy findings, we first examined the FamCare*Plus* application usage logs with login data of the experimental group participants. Table 5 summarizes these details, showing the total number of days each participant actively used the application, the total number of times they accessed the application, and the total number of days the application was installed. For example, participant 7 had the application for 30 days and used it on 14 different days for a total of 40 sessions, indicating high engagement. In contrast, participant 6 had the application for 25 days but only used it for 5 days with 11 sessions, reflecting lower activity. Overall, the data reveal varying engagement levels among participants in the experimental group. Using the Pearson correlation coefficient (r), we found no correlation between application usage and AD levels among the experimental group. These results, however, may be due to the small sample size.

A comparison of AD levels between family members who received the mHealth intervention and those who did not during the 3-week study revealed notable differences from Week 1 at baseline and post-study outcomes (Table 6).

Compared to baseline mean scores, the experimental group reported lower AD levels at Week 1 (41.9% and 27.8%, respectively) than the control group (55.2% and 34.2%). From Week 1 to Week 3, the percentage change shows an 8.6% decrease in anxiety in the experimental group, compared to a 12.8% decrease in the control group. Although anxiety levels in the control group decreased more than those in the experimental group after 3 weeks, this may be because the experimental group started with lower anxiety at Week 1, leaving less room for further reduction. However, in Week 3, depression decreased by 8.1% in the experimental group versus 5.7% in the control group.

After analyzing the mean scores, we conducted a Mann–Whitney U test to examine potential differences between and within groups. Given the small sample size, no statistically significant differences were detected at the *p* < 0.05 level. As the next step, we analyzed the data applying the HADS scoring guidelines. This analysis included 2 subscales (HAD-A for anxiety and HAD-D for depression), with a maximum score of 21. According to these guidelines, scores of 0–7 are classified as Normal, 8–10 as Borderline Abnormal (mild symptoms), and 11–21 as Abnormal (clinically significant symptoms).

Our findings showed that in Week 1, the control group exhibited anxiety levels in the Abnormal range (significant symptoms according to the HADS clinical criteria), whereas the experimental group was in the Borderline range (mild symptoms). For depression, the control group fell within the Borderline category, whereas the experimental group was Normal. These results indicate that participants receiving the intervention started the study with better AD profiles compared to those in the control group. In Week 3, the control group improved from Abnormal to Borderline for anxiety, whereas the experimental group progressed from Borderline to Normal. Regarding depression, the control group moved from Borderline to Normal, whereas the experimental group, already in the Normal range, showed further improvement (see Table 7).

Overall, these results suggest that the mHealth intervention contributed to maintaining and enhancing mental health outcomes, with the experimental group consistently demonstrating superior improvements compared to the control group (Table 7).

## 4. Discussion

In the present study, we aimed to investigate the feasibility, usability, and efficacy of the mHealth application FamCare*Plus* with family members of critical-care patients admitted to the BMT unit in the MCC at the University of Kentucky. Cancer patients who are admitted for treatment for 3 weeks or more have a life-threatening medical condition that places a family member in a psychological condition of extreme uncertainty, accompanied by high levels of AD. Adding to this condition, research has consistently demonstrated for over a decade that family members suffer in part because of a lack of updates and communication from the bedside staff. In response to these challenges, the researchers developed the mobile application FamCare*Plus*.

As such, we hypothesized that after 3 weeks of using the FamCare*Plus* intervention, participants in the experimental group would demonstrate feasibility for an intended future study, that the application had a high degree of usability, and that participants would report lower AD scores than the control group.

Our ability to recruit family members of BMT patients within weeks of treatment and close observation demanded that the researchers exercise sensitivity to the mental health condition of the patient and their family members. After obtaining Institutional Review Board (IRB) approval, we began the recruitment and consent process. As noted, throughout the 8 months of recruitment and data collection, only one family member from the experimental group withdrew from the study because of personal issues. Their withdrawal was recorded in the withdrawal log document and reported to the IRB.

Regular contact with participants throughout the 3 weeks resulted in no major complications with the use of the application. We also observed no adverse events from any family member in their use of the mobile application. In sum, we met our feasibility criteria of a > 80% threshold of participants completing the study. The feasibility of a future study with a larger sample is promising, given our low attrition rate (5%) and high enrollment rate (25%), which also met our a > 80% threshold for recruitment.

We also observed that all SUS and NASA scores were in the higher index, suggesting a high degree of usability, with low workload demand on participants. As noted, we analyzed SUS scores in two ways. Raw score ratings for all participant family members for each question, with an overall mean score of 4.20 (SD = 0.84) and a total mean SUS score of 80 (SD = 21.02), both above averages, indicating a high degree of usability. Regarding the NASA survey measures, we observed a mean score of 27.50, also well below 40, suggesting a low demand for workload tasks while using the intervention.

Regarding efficacy, the findings of this study demonstrate that the use of the FamCare*Plus* mHealth intervention was associated with a sustained reduction in AD levels among family members of patients admitted to BMT. Over the 3 weeks, family members who received the mHealth intervention experienced lower AD compared to those who did not. The reduced psychological burden in the experimental group, both at the first week and by the end of Week 3, may be attributed to daily patient health updates and the continuous connection with the bedside provided by the intervention. Over the 3-week study, anxiety decreased in both groups, though the group without the intervention showed a larger drop, likely because the intervention group started lower. Depression decreased more in the experimental group than in the control group by the end of the study.

These trends were further validated when we analyzed the scores according to HADS guidelines, with ranges from Abnormal to Borderline to Normal. The comparative analysis of AD scores between the experimental and control groups highlights the potential benefits of the FamCare*Plus* intervention. At baseline, families using the intervention exhibited milder symptoms, with anxiety in the borderline range and depression within normal limits, whereas the control group presented with clinically significant anxiety and borderline depression. By Week 3, both groups improved; however, the experimental group progressed to normal levels for anxiety and maintained normal depression scores with further improvement, whereas the control group only moved from abnormal to borderline for anxiety and from borderline to normal for depression.

These trends suggest that the mHealth intervention not only helped sustain mental health stability but also enhanced outcomes compared to traditional communication methods. The continuous access to patient updates and engagement with bedside care likely contributed to reducing uncertainty and emotional distress, reinforcing the value of family-centered digital tools in critical-care settings.

These results align with prior research indicating that timely, consistent communication between health care providers and patients’ families can alleviate psychological distress [11,12,13,14,15,16,90]. FamCare*Plus* appears to have achieved this by facilitating direct, continuous updates and engagement with medical staff, thereby enhancing trust and perceived involvement in care decisions—factors known to reduce uncertainty and emotional strain [11]. The study sample, drawn from family members of stem cell transplant patients, presents a particularly relevant group because these individuals often face prolonged critical-care stays, complex prognoses, and heightened emotional burden [91].

An important consideration is that the study focused on families residing in remote areas, for whom physical access to the hospital and medical staff was challenging. This subgroup stands to benefit substantially from mHealth solutions, which can bridge geographic barriers and ensure timely communication. The observed reductions in AD within this population underscore the potential of digital interventions to address disparities in health care access and psychosocial support [43,44,92].

Overall, this study provides preliminary evidence supporting the feasibility and potential benefits of FamCare*Plus* as a tool for reducing psychological distress among ICU patient families, particularly in remote locations. Although the absolute percentages may appear modest, such changes are clinically meaningful, particularly in high-stress environments such as critical-care settings, where even small improvements in mental health can have significant implications for well-being and coping capacity [93]. The integration of mHealth interventions into critical-care practice holds promise for improving both family and patient outcomes and merits further rigorous investigation.

## 5. Conclusions

This study provides preliminary evidence that the FamCare*Plus* mHealth intervention may reduce AD among family members of critical care patients residing in remote areas, with notable improvements observed from the first week and sustained over 3 weeks. Endpoint results suggest that such digital tools may bridge communication gaps and improve psychological well-being in underserved populations living in rural locations in the United States and worldwide. Although the small sample size limits generalizability, these findings highlight the potential of mHealth solutions to enhance family support in critical care and warrant further research in larger and more diverse patient populations and in other care facility types.

## 6. Limitations

This study has several limitations. First, as a quasi-experimental pilot study, our findings resulted in a small overall sample size. The 9 and 10 participants in the experimental and control groups, respectively, limit the generalizability of the findings for indicating statistical differences. Also, the team noticed that the experimental group had a higher level of variability (larger standard deviation of the total scores) in both AD measures, which may have contributed to the lack of statistical significance. The study followed participants at Week 3, which may not be sufficient to capture the long-term effects of FamCare*Plus* on AD. Also, because participants and researchers were not blinded, we acknowledge the risk of bias. As such, our mitigating analyses have resulted in an adjustment of baseline expectations.

Despite these limitations, this study provides valuable insight into the potential benefits of using FamCare*Plus* in reducing AD in families of BMT, ICU, or other critical care patients. Future studies with larger sample sizes and possibly longer follow-up periods are needed to further investigate this novel intervention. Using a convenience sample, participants were more readily accessible to the researcher, creating opportunities that were not equal for all qualified individuals in the target population. As such, the results are not necessarily generalizable to this population.

## 7. Future Research

Although these findings are promising, the relatively small sample size limits the generalizability of the results. Larger, multicenter trials should be conducted to validate these outcomes across different patient populations, such as families of patients with cardiac, neurological, pulmonary, or trauma-related critical illness or those in the general ward of a hospital. The study could also be replicated with patients at different life stages because factors such as age or proximity to end-of-life may influence how families respond to this intervention [94]. Furthermore, longitudinal studies are warranted to explore whether improved family mental health through mHealth interventions may indirectly contribute to patient recovery because the psychological well-being of family members has been linked to improved care collaboration and decision-making in the ICU.

## Figures and Tables

**Table 1:** Comparability demographic table of all participants.

Demographic		Experimental Group	Control Group
(*n* = 19)		(*n* = 9)	(*n* = 10)
Gender	Female	8 (88.88%)	8 (80.00%)
	Male	1 (11.12%)	2 (20.00%)
Age	20–29 yrs.	1 (11.10%)	N/A
	40–49 yrs.	3 (33.30%)	3 (30.00%)
	50–59 yrs.	1 (11.10%)	3 (30.00%)
	60–69 yrs.	2 (22.20%)	1 (10.00%)
	70–79 yrs.	2 (22.20%)	3 (30.00%)
Ethnicity	White	8 (88.88%)	9 (90.00%)
	Black	1 (11.12%)	N/A
	Hispanic/Latino	N/A	1 (10.00%)
Travel Distance from Home to Markey BMT	5 to 25 miles	N/A	3 (30.00%)
	26 to 50 miles	4 (44.44%)	1 (10.00%)
	51 to 100 miles	4 (44.44%)	3 (30.00%)
	101 to 250 miles	N/A	3 (30.00%)
	251 to 500 miles	1 (11.12%)	N/A

**Table 2:** Results of the System Usability Scale questionnaire. Total score range: 0–5, including the collective (*n* = 7) mean and standard deviation per question.

SUS Survey Questions	Mean (SD)
Q1. I think that I would like to use this system frequently.	3.86 (1.46)
Q2. I found the system unnecessarily complex.	4.00 (1.53)
Q3. I thought the system was easy to use.	4.43 (0.53)
Q4. I would need the support of a technical person to be able to use this system.	3.71 (1.70)
Q5. I found that the categorized results in this system were very sensible.	4.14 (1.21)
Q6. I thought that there was too much inconsistency in this system.	4.57 (0.79)
Q7. I would imagine that most people could learn to use this system very quickly.	4.43 (0.98)
Q8. I found the system very cumbersome to use.	3.86 (1.21)
Q9. I felt very confident using the system.	4.29 (1.25)
Q10. I needed to learn a lot of things before I could get going with this system	4.71 (0.76)
Final SUS Score	4.20 (0.84)

**Table 3:** Results of the System Usability Scale questionnaire. Total score range: 0–100, including the collective mean score per question, with the total score and standard deviation.

	Q1	Q1	Q2	Q3	Q4	Q5	Q6	Q7	Q8	Q9	Q10	
Participants	2	4	4	3	4	4	4	4	4	4	4	98
	3	4	4	4	4	4	4	4	4	4	4	100
	4	3	4	4	4	3	4	4	3	4	4	93
	5	2	0	4	4	4	4	4	2	4	4	80
	6	0	2	3	0	1	4	2	1	1	4	45
	7	4	4	3	1	4	3	4	4	4	4	88
	8	3	3	3	2	2	2	2	2	2	2	58
Final SUS Score												80 (SD = 21)

Roll sum multiplied by 2.5 to arrive at a final score according to the SUS method.

**Table 4:** Results of the NASA-task load index questionnaire. Total score range: 0–100.

Ques.	Demand Type	Question	Mean (SD)
Q1. Mental How mentally demanding was the task?			37.14 (19.76)
Q2. Physical How physically demanding was the task?			28.57 (35.20)
Q3. Temporal How hurried or rushed was the pace of the task?			28.57 (35.08)
Q4. Performance How hard did you have to work to achieve your level of performance?			23.57 (22.12)
Q5. Effort How successful were you in accomplishing what you were asked to do?			30.00 (31.36)
Q6. Frustration How insecure, discouraged, irritated, stressed, and annoyed were you?			17.14 (13.80)
Final NASA-TLX Score			27.50 (15.45)

**Table 5:** Results of the FamCare*Plus* app usage logs for the experiment group.

Participants	No. of Different Days Using the App	No. of Times Using the App	Total Days Having App
1	12	36	43
2	8	23	34
3	7	33	25
4	10	32	38
5	7	20	38
6	5	11	25
7	14	40	30
8	6	20	21
9	12	18	26

**Table 6:** Comparison of mean anxiety and depression scores between experimental and control family groups, weeks one through three.

Mean Anxiety and Depression Scores: Comparing Week 1 (Baseline) to Week 3 (Post Study)								
	Control Group (*n* = 10)			Experimental Group (*n* = 9)			Difference Between Groups	
	Week 1 M (SD)	Week 3 M (SD)	Within Group Δ	Week 1 M (SD)	Week 3 M (SD)	Within Group Δ	Week 1	Week 3
Anxiety	11.6 (4.8) 55.2%	8.9 (5.9) (42.4%)	(55.2–42.4) 12.8%	8.8 (2.8) 41.9%	7.0 (5.6) 33.3%	(41.9–33.3) 8.6%	(55.2–41.9) 13.3%	(42.4–33.3) 9.1%
Depression	7.2 (4.1) 34.2%	6.0 (4.1) (28.5%)	(34.2–28.5) 5.7%	5.7 (3.6) 27.2%	4.1 (4.9) 19.1%	(27.2–19.1) 8.1%	(34.2–27.14) 7%	(28.5–19.1) 9.4%

**Table 7:** Comparison of anxiety and depression scores as per HADs guidelines.

	Control Group		Experimental Group	
	Week 1 (Scores)	Week 3 (Scores)	Week 1 (Scores)	Week 3 (Scores)
Anxiety	Abnormal (11.6)	Borderline (8.9)	Borderline (8.8)	Normal (7.0)
Depression	Borderline (7.2)	Normal (6.0)	Normal (5.7)	Normal (4.1)

Note: HADs Score: 0–7 Normal, 8–11 Borderline, 12–21 Abnormal.

## Data Availability

The original contributions presented in this study are included in the article. Further inquiries can be directed to the corresponding author.

## Abbreviations

The following abbreviations are used in this manuscript:

AD	Anxiety and depression
BMT	Bone marrow transplant
EMR	Electronic medical record
HADS	Hospital anxiety depression scale
ICU	Intensive care unit
MCC	Markey Cancer Center
SUS	System Usability Scale
